# Research protocol for bridging research, accurate information and dialogue (BRAID)—clinical trials: a mixed-methods study of a community-based intervention to improve trust and diversify participation in clinical trials

**DOI:** 10.3389/fpubh.2024.1407726

**Published:** 2024-09-16

**Authors:** Damara N. Gutnick, Patricia Lozano, Smeily Rodriguez Martinez, Katherine W. Wang, Debra A. Williams, Bruce D. Rapkin, Nelly Gonzalez-Lepage

**Affiliations:** ^1^Department of Epidemiology and Population Health, Albert Einstein College of Medicine, Bronx, NY, United States; ^2^Department of Family and Social Medicine, Albert Einstein College of Medicine, Bronx, NY, United States; ^3^Department of and Psychiatry and Behavioral Sciences, Albert Einstein College of Medicine, Bronx, NY, United States; ^4^Insititute for Clinical and Translational Research, Albert Einstein College of Medicine, Bronx, NY, United States; ^5^Office of Community and Population Health, Montefiore Medical Center, Bronx, NY, United States; ^6^Department of Child and Adolescent Psychiatry, NYU Langone Medical Center, New York, NY, United States; ^7^NYC Health + Hospitals/Bellevue Hospital, New York, NY, United States

**Keywords:** community based participatory research, community engagement, trusted messengers, diversity in clinical trials, trust in science, vaccine hesitancy, health equity, motivational interviewing

## Abstract

Cultural beliefs, personal experiences, and historic abuses within the healthcare system—rooted in structural racism—all contribute to community distrust in science and medicine. This lack of trust, particularly within underserved communities, contributes to decreased participation in clinical trials and a lack of representation in the data. Open dialogue about community concerns and experiences related to research participation and medical care processes can help build trust and change attitudes and behaviors that affect community health. This protocol outlines an approach to increase trust in science and clinical trials among communities in the Bronx, New York that are typically underrepresented in research data. Bridging Research, Accurate Information and Dialogue (BRAID) is a two-phased, evidence-based community engagement model that creates safe spaces for bilateral dialogues between trusted community messengers, and clinicians and scientists. The team will conduct a series of BRAID Conversation Circles on the topic of clinical trials with local trusted community messengers. Participants will be members of the community who are perceived as “trusted messengers” and can represent the community’s voice because they have insight into “what matters” locally. Conversation Circles will be audiotaped, transcribed, and analyzed to identify emergent challenges and opportunities surrounding clinical trial participation. These key themes will subsequently inform the codesign and co-creation of tailored messages and outreach efforts that community participants can disseminate downstream to their social networks. Surveys will be administered to all participants before and after each Conversation Circle to understand participants experience and evaluate changes in knowledge and attitudes about clinical trials, including protections for research participants the advantages of having diverse representation. Changes in motivation and readiness to share accurate clinical trial information downstream will also be assessed. Lastly, we will measure participants dissemination of codesigned science messages through their social networks by tracking participant specific resource URLs of materials and videos posted on a BRAID website. This protocol will assess the effectiveness and adoptability of an innovative CBPR model that can be applied to a wide range of public health issues and has the potential to navigate the ever-changing needs of the communities that surround health systems.

## 1 Introduction

In order to meaningfully engage communities to co-create and implement strategies to achieve health equity and combat misinformation, anchor institutions need to first earn trust. Many health disparities are rooted in historic structural racism (1, 2). Repeated injustices, including a legacy of research abuses and medical exploitation of underserved communities, have reinforced distrust in medicine and research within communities of color (3, 4).

To date, individuals in the United States identifying as Black or Latino represent 15 and 13% of those affected by cancer, respectively, yet only comprise 4–6% and 3–6% of those enrolled in clinical trials (5). Since the onset of the COVID-19 pandemic, barriers to the participation of underrepresented populations in research have persisted, potentially deepening healthcare inequities and limiting patient access to precision medicine (6–8). Though the 1993 National Institutes of Health Revitalization Act mandated an increase in the enrollment of women and underrepresented ethnic groups in clinical trials, efforts to combat underrepresentation by research teams have had limited success (9, 10). Therefore, there has been a continued and evolving effort by the National Institutes of Health to increase the participation of underrepresented populations in clinical trial research.

A systematic review of published interventions aiming to increase the participation of underrepresented populations in cancer clinical trials described efforts ranging from patient navigators, culturally-tailored informational videos, research team competence training, and relationship building/social marketing (5). While many of the efforts demonstrated favorable increases in enrollment of underrepresented populations, it is difficult to conclude that one particular approach is most impactful, as achieving diversity in clinical trials is a multifaceted issue. However, research has consistently demonstrated that trust and a feeling of partnership with members of the medical community are key influencers of patient willingness to participate in clinical trials (4, 11, 12).

Since communities shift over time and can vary greatly among each other, using a community-based engagement model to elicit rapid input on perceptions, doubts, and knowledge gaps poses a promising way to strengthen trust and determine best practices for working with individual communities. To narrow the gap of existing health disparities, distrust in scientific research and healthcare delivery systems must be addressed. The creation of safe spaces for open, bidirectional conversations, where communities can share their experiences and expectations, can help rebuild and nurture trust, and form a foundational infrastructure to meaningfully move toward health equity (13).

Montefiore/Einstein is the largest healthcare provider in the Bronx, New York. The borough is home to a large community of color, with 44 and 57% of the population identifying as Black or African American, and Hispanic or Latino, respectively (14). To build trust in science, medicine, and research (e.g., clinical trials) in the Bronx communities, our research team will employ the Bridging Research, Accurate Information and Dialogue (BRAID) model to engage trusted community messengers from the Bronx in a series of ongoing dynamic dialogues called Conversation Circles (15–19).

We anticipate that within these safe spaces, community concerns related to trust in research, science, healthcare, structural racism, and health disparities will emerge, and gaps in knowledge and misinformation can be identified and filled. As accurate information is shared within the circles, our team will facilitate codesign processes to support the coproduction of accurate scientific messages tailored to the local community. We predict that participation in BRAID will enhance community confidence and trust in science and clinical trials, as well as increase trusted community messenger motivation and self-efficacy to disseminate accurate information (co-created messages) through their social networks.

## 2 Methods and analysis

### 2.1 The BRAID model

**Figure 1:**
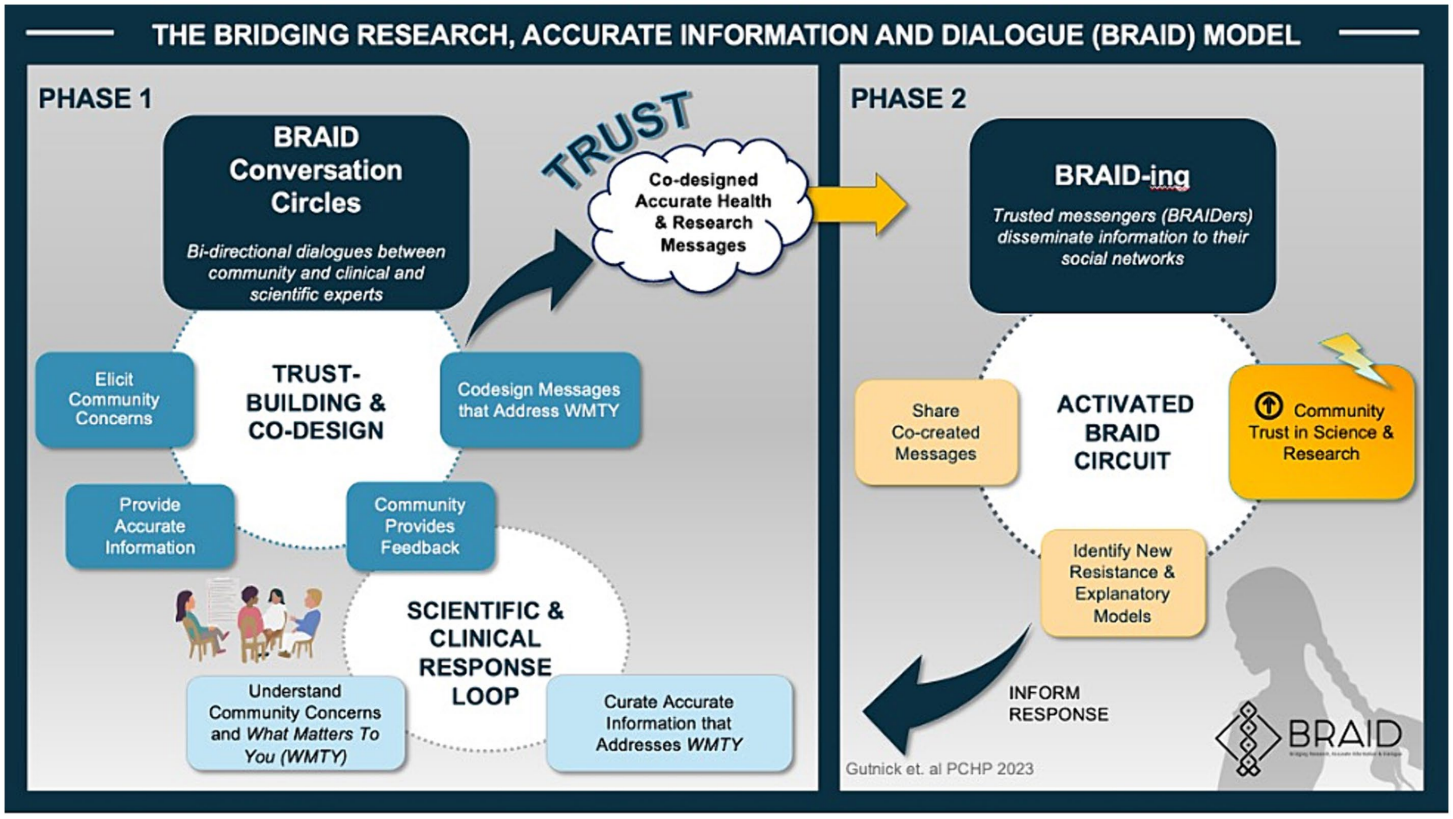
The bridging research, accurate information and dialogue (BRAID) model.

BRAID is an evidence-based, iterative, biphasic community engagement model developed by author D.G and researchers at the Albert Einstein College of Medicine (15–19). The model closely aligns with the Association of American Medical Colleges Principles of Trustworthiness (13, 15) and is summarized in Figure 1.

**Figure 2:**
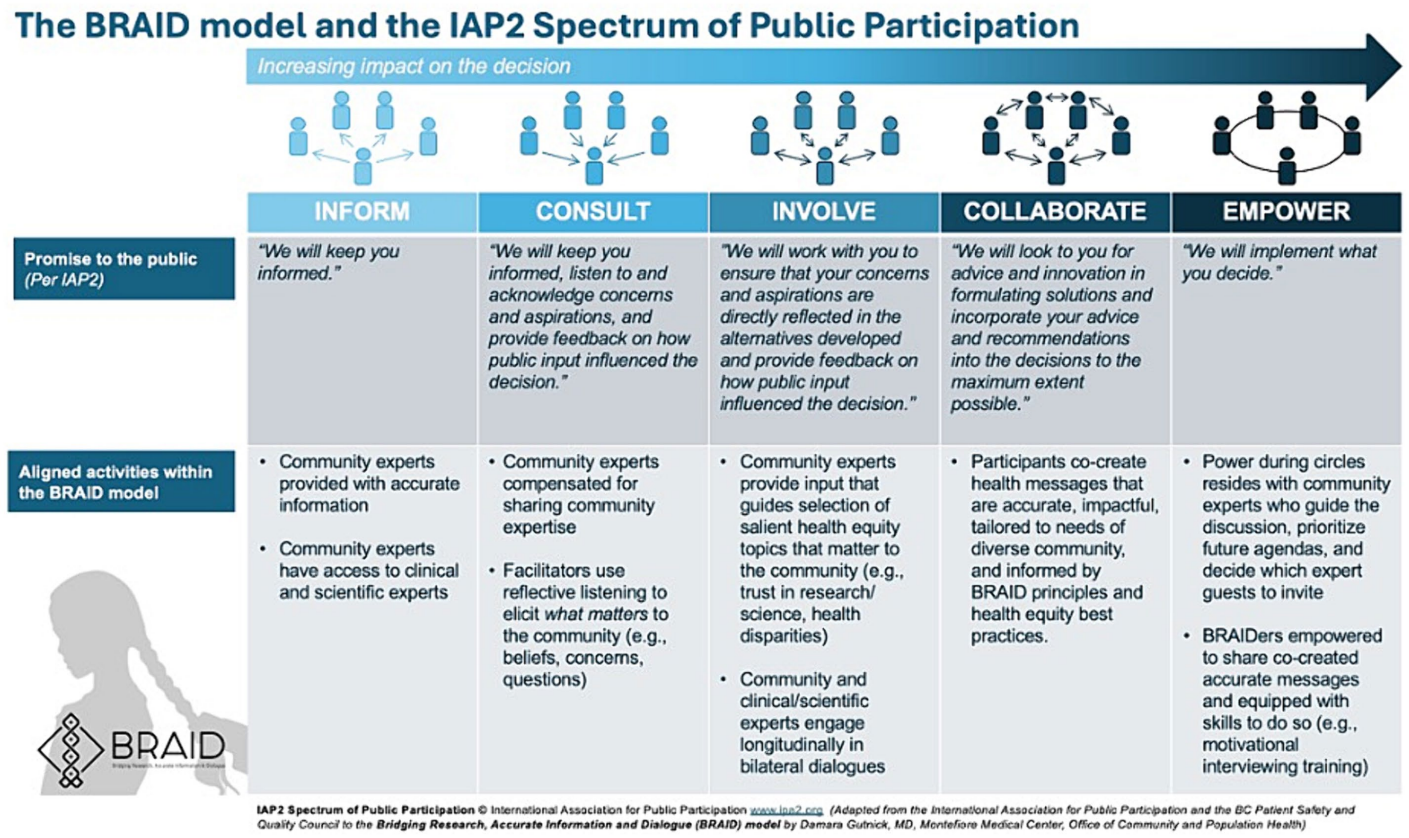
The BRAID model aligns closely with the IAP2 spectrum of public participation (29, 30).

During Phase 1 of the BRAID model, safe spaces (“Conversation Circles”) are created for ongoing dialogues between “trusted community messengers” (“Community Experts”) and clinicians and scientists representing public health and/or health systems. Once a sense of trust is established, participants are supported as they work together to co-create accurate health messages tailored for the local community. In Phase 2 of the model, trusted messengers are empowered to disseminate co-created messages through their social networks. The process of sharing information is called “BRAIDing” and the trusted community messengers who share information are called “BRAIDers.” BRAIDers bring lessons learned about emerging community concerns and beliefs (i.e., conspiracy theories, misinformation) back to the Conversation Circles to inform the development of new health messages that address *what matters* to their community. This iterative approach moves community members along the spectrum of public participation (Figure 2). A glossary of key Conversation Circle terms can be found in Appendix A and a BRAID implementation toolkit including guidance for implementing BRAID with fidelity is in development.

BRAID is adoptable to a wide range of public health issues and has the potential to navigate the ever-changing needs of the communities that surround health systems (15). Previously implemented by the team to elicit community perceptions and hesitations related to the COVID-19 vaccine, BRAID was successful in achieving robust community engagement and building trust (20).

**Figure 3:**
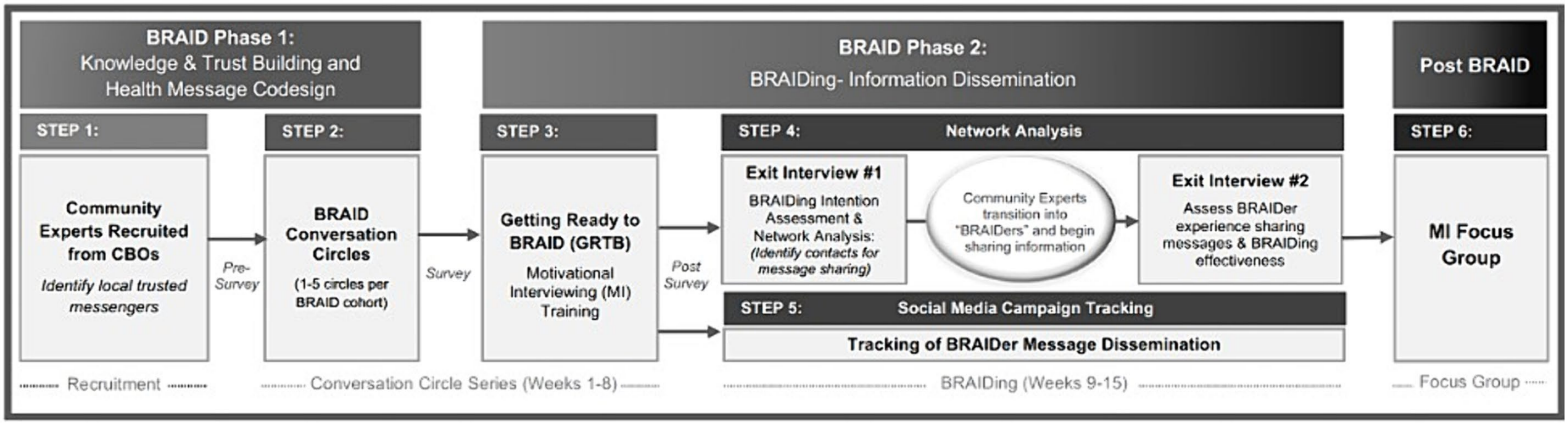
BRAID study design.

Our procedures for this BRAID mixed-methods study were adapted from the Community Engagement Studios Manual developed by Vanderbilt’s and Meharry’s Clinical and Translational Science Award community engagement core (21). Figure 3 outlines how our study design aligns with the biphasic BRAID model. Each of the panels in the figures are described in detail in the proceeding sections:

### 2.2 BRAID phase 1: knowledge and trust building and health message codesign (steps 1–2)

#### 2.2.1 Identifying trusted community messengers (step 1)

We will first partner with local community-based organizations (CBOs) to identify and engage trusted community messengers (“Community Experts”), well-connected and influential individuals from target communities who represent the communities’ voice. In the BRAID model, CBOs able to identify and help recruit trusted messengers are called “BRAID Strands.” Natural BRAID Strands for this effort include but are not limited to faith-based groups, senior centers, schools, tenants’ councils, libraries, museums and cultural institutions, federally qualified health centers, clinics, therapy groups and food assistance programs. These organizations are pillars of the community, with their members typically having robust social networks. This makes them ideal candidates for the BRAID trusted messenger role. We will compensate our partnering BRAID Strands for performing administrative tasks as outlined in Appendix B. Aligned with the best practices of community-based participatory research (CBPR), individual Community Experts will be compensated for their participation in Conversation Circles. Compensation will reflect the standards established by PCORI-funded research guidelines (22).

#### 2.2.2 BRAID Conversation Circles (step 2)

Once trusted messengers are identified, we will invite them to participate in a series of dynamic BRAID “Conversation Circles.” During these dialogues, a motivational interviewing (MI) aligned facilitation style will be employed to elicit important community perspectives, understand “*what matters*” most to the community, and create a space where clinical and scientific experts are invited to share accurate information that addresses community concerns (23). We envision that themes related to trust in research, science, healthcare, structural racism, and health disparities will emerge, and that gaps in knowledge and misinformation will be identified. Further, Conversation Circles will be similarly used to obtain community feedback and sentiment on published research findings directly relevant to members in their community. Throughout the Conversation Circle process, a facilitator will guide the participants towards critically reviewing learned information and codesigning solutions and accurate messages that they deem appropriate to share with their community networks.

We will implement up to 15 unique Conversation Circle cohorts. Four to 12 trusted community messengers will participate in each cohort, and clinical and scientistic guests will be selected and invited to attend and contribute to the dialogue based on their ability to address emerging community concerns. Each Conversation Circle will meet 1–5 times with 1–3 weeks between each circle. Conversation Circles will take place in-person or virtually using Zoom and will be recorded and transcribed. Surveys will be collected before and after each Conversation Circle to understand participant experience and confidence in knowledge of accurate information, assess participant perception of the information shared (i.e., the benefit of having diversity in clinical trials, protections in place to protect study participants, relevant study findings), and explore participant desire and self-efficacy to share accurate information downstream to their community (Appendix C).

### 2.3 BRAID phase 2: information dissemination (BRAIDing; step 3–4)

#### 2.3.1 Getting ready to BRAID: motivational interviewing training (step 3)

We anticipate that teaching BRAIDers Motivational Interviewing (MI) based communication skills will strengthen their confidence and comfort in sharing co-created educational messages that may be controversially perceived by their community (i.e., vaccine and clinical trial messages). Participants will therefore be invited to participate in a 60–90 min “Getting Ready to BRAID” session which will introduce MI spirit and skills including reflective listening, and the ASK-TELL-ASK tool for sharing information and advice. The curriculum will be tailored to incorporate guidance on how to respond to the specific types of resistance statements that Community Experts encounter.

#### 2.3.2 Assessing readiness to BRAID: exit interviews (step 4)

Following the “Getting Ready to BRAID” session, a structured interview with each BRAIDer will be conducted to assess their BRAIDing comfort and intention. BRAIDers will be asked a series of questions that align with the PROSCI™ Change Management ADKAR (Awareness, Desire, Knowledge, Ability and Reinforcement) model, and their responses will be recorded on a scale from 1–5 (1 = “Not At All,” 5 = “Extremely”; Appendix D) (24). Interview answers will be collected in a SurveyMonkey form by the research assistant. These responses will be used to identify factors influencing BRAIDers’ readiness for sharing information throughout their social networks, and for responding to individuals’ needs for further training and resources to improve their readiness.

To assess BRAIDers experience and effectiveness in sharing information, we will also conduct a network analysis beginning in the first exit interview. This will involve first asking each BRAIDer to identify up to 8 individuals (e.g., family, friends, coworkers) with whom they intend to share the co-created messages. For each of these individuals, we will collect their initials, demographic information, relationship to the BRAIDer, and the BRAIDer’s prediction of the individual’s anticipated reaction to the messages. BRAIDers will be provided with materials introduced during the Conversation Circles, including FAQs, websites, and videos, presented in formats that are easily shareable with others. After 3–6 weeks, each BRAIDer will complete a second exit interview to discuss their experience sharing information and provide feedback on how their chosen community members responded to the messages shared.

#### 2.3.3 Tracking BRAIDer message dissemination through social media (step 5)

In addition to the structured interviews, for our network analysis we will also monitor BRAIDer message dissemination through social media. Codesigned messages, video links, and other clinical trials materials and resources will first be posted on a dedicated BRAID website. In order to monitor engagement all website links will feature Google analytics campaign tracking. When a link is copied and shared by a BRAIDer, their unique alpha-numeric campaign code (which will not include identifying information) will be appended to the resource’s URL. This will enable us to monitor which BRAIDers are responsible for a given share of website traffic.

### 2.4 Post BRAID: focus group (step 6)

After BRAIDers personally gain BRAIDing experience, they will be invited to participate in a focus group designed to understand BRAIDers experience sharing information, whether they felt that learning MI skills was valuable, and if they utilized these skills during the BRAIDing process. The insights gathered from these focus groups will support the opportunity to collaborate and co-design acceptable health messages with community experts, reflecting *what matters* most to them.

### 2.5 Data analysis plan

BRAID is a mixed-methods study. Key quotes and themes from the transcriptions of the audio or video-recorded Conversation Circles will be identified via inductive thematic analysis based on a codebook that will be developed by the BRAID research team. At least 2 BRAID research team members will independently code each Conversation Circle transcript using Dedoose qualitative analytics software. Following initial Conversation Circle transcript coding, the BRAID team will collectively assess inter-coder reliability and alignment of transcript coding to the established code book categories. Network analysis will be conducted using data collected from the “Getting Ready to BRAID” interview responses to identify associations between BRAIDer responses and successful BRAIDing. Descriptive statistics and quantitative data from both pre- and post-Conversation Circle survey responses will be analyzed using SPSS and/or R. All data will be analyzed using grounded theory methods (25, 26). Google campaign analytics will be used to track the reach of each BRAIDer’s message dissemination through social media.

## 3 Discussion

For members of historically underrepresented communities, fostering trust in research and science is vital to increasing their participation in research projects. By respecting the community voice through engagement with trusted messengers and incorporating codesign principles, BRAID may be able to foster community trust among members who might otherwise be difficult to reach. In fact, the BRAID model has already been shown to foster rich dialogue regarding COVID-19 vaccine hesitancy in the Bronx, and has also demonstrated its ability to empower trusted messengers (BRAIDers) to share accurate information about vaccine safety (20).

We anticipate that the BRAID model could be rapidly implemented with the help of diverse community partnerships to identify health disparities that exist across socioeconomic status, age, geography, gender, disability status, sexual identity and orientation, citizenship status, and more. Furthermore, as a community engagement model, BRAID can be readily adapted to extend beyond increasing clinical research participation. We envision that the model could potentially be used to build trust, co-design and disseminate accurate health messages, and obtain community feedback on health and social care issues that drive health disparities including, but not limited to, vaccination status, food insecurity, mental healthcare, and cancer prevention.

Our study has several limitations. First, recruitment bias may be introduced because our CBO partners will be responsible for identifying potential Community Experts rather than community members volunteering themselves or being selecting by the BRAID research team. We attempted to minimize this bias by creating clear guiding principles for objectively identifying BRAID participants which will be shared with our CBO partners and included in our BRAID manual. Our protocol is also potentially subjective to social desirability bias. While it is important to recognize that the goal of BRAID Conversation Circles is to provide a space for bidirectional dialogues that may influence or altogether change the attitudes and beliefs of participants, we will distribute pre- and post-surveys outside of the Conversation Circle group setting to minimize this risk. As the process of engaging with participants who do not complete their series of Conversation Circles is not included in the current protocol, potential for attrition bias also exists. Lastly, we acknowledge that our study findings may not be clearly generalizable to communities different than the ones included within the Conversation Circles. Nevertheless, the BRAID model can be adapted to address a broad range of public health concerns regardless of community demographics.

BRAID has the potential to serve as a foundational infrastructure for a Learning Health Care Community (27, 28). In this paradigm, a robust network of “BRAIDers” who trust science and the healthcare system, could be leveraged by an anchor institution to provide ongoing local community input into health equity programming, research design, and dissemination. This kind of long-term investment can further cultivate meaningful community partnerships and collaborations that address the underlying structural factors that drive health disparities, in order to achieve health equity.
