# Supplementary material for: Research protocol for bridging research, accurate information and dialogue (BRAID)—clinical trials: a mixed-methods study of a community-based intervention to improve trust and diversify participation in clinical trials
Source: Front Public Health. 2024 Sep 16;12:1407726. doi: 10.3389/fpubh.2024.1407726 (PMC11439785; doi:10.3389/fpubh.2024.1407726)

**Appendix A**

**Glossary of Key Conversation Circle Terms**

Here are a few terms (below) that are used to describe the BRAID Conversation Circle roles, activities, and elements. These terms are referred to and explained in context in subsequent sections of this guide as well as in the facilitator guide.

**Community Experts:** Well-connected and influential individuals from target communities who are trusted messengers or influencers and participate in BRAID Conversation Circles. These individuals represent the communities’ voice. As experts with insight into *“what matters”* most to their communities, during the dialogues they are encouraged to share prominent beliefs, concerns, questions or conspiracy theories circulating locally within their social networks. Community experts who share information downstream through social networks are called “BRAIDers.”

**Conversation Circle**: A group of 4-12 Community Experts convened to participate in an open discussion about issues that matter to them and their communities. Circles may also include Clinical and Scientific Experts and are guided by a Circle Facilitator.

**BRAID Facilitator:** A person who encourages and enables an open and balanced discussion between participants (Community Experts/Scientific Experts) during the Conversation Circle. BRAID facilitators utilize reflective listening skills to draw out *what matters most* to participants. At the conclusion of each conversation circle, the Facilitator guides a prioritization exercise to help Community Experts decide the agenda topics for the next follow up circle and which clinicians and scientists to invite.

**BRAID Support Team:** Person/’s providing logistical support for the Circle Facilitator and participants during the circle, including note taking, data collection, managing the chat, and other facilitation support.

**BRAIDer:** A Community Expert who co-creates and shares accurate and relevant information with their in-person or social media networks**.** The goal is for Community Experts to become BRAIDers.

**Clinical/Scientific Experts:** Health professionals, researchers, and other experts with the knowledge, skill, and training required to answer subject matter-related questions, who are invited to the circle based on information requested by Community Experts.

**Braid Strands:** Community partners, such as Community or Faith-Based Organizations, health centers, schools, or other organizations who agree to facilitate connections to members for BRAID circles.

**Parking Lot:** A running list of questions or concerns raised by Community Experts during the circle to be addressed at a later date by Clinical/Scientific Experts, data, or information. The list is created using a blank sheet or page (for virtual meetings) and is shared with participants at the close of the session.

**Parking Lot Attendant:** The BRAID team member who maintains the parking lot during the dialogue by capturing key themes, questions, and community expert requests as they arise. At the conclusion of each circle, the Parking Lot Attendant briefly summarizes key themes back to the group.

**Informed Consent:** (If conducting BRAID as a research study) The process of explaining the study purpose, procedures, and terms of participation to prospective participants before obtaining their verbal or written consent to participate.

**Appendix B**

**Pre- and Post- Conversation BRAID Circle Feedback Forms**

**PRE-CONVERSATION BRAID CIRCLE FEEDBACK FORM (Administer before Session #1)**

Name

Date

***A researcher or scientist is someone who does research to investigate disease and test ways to prevent disease and treat disease***[NG1]

**1.**  **When you hear the term “medical research”, what comes to mind?** _____________

**2.** **Have you ever been asked to participate in a medical research study?**

a. Yes **→** SKIP to 2ai

b. No **→** SKIP to 2b

c. Not sure **→** SKIP to 2b

**2ai. Did you participate in any research study**?

a. Yes àSKIP to 2aii

b. No, I wanted to participate but didn’t meet study criteria à SKIP to 2b

c. No, I did not want to participate à SKIP to 2b

*If yes, to 2ai then* **→**

**2aii.** **What kinds of research studies did you participate in? (Check all that apply)**

a. Clinical trial for testing new medications or therapies

b. Interview with a researcher

c. Focus Group (Several people interviewed as a group)

d. Survey – paper or electronic format

e. Telephone opinion poll

f. Other: __________

*if no to 2 or 2ai then* ***→***

**2b. We are interested in understanding people’s attitudes about clinical trials. Clinical trials are studies where new medical treatments or therapies are tested.**

***Let’s say that you have a medical condition, and there is a clinical trial to test a new treatment that may or may not be better than current treatments for the health condition you have****.*

**If you were asked today, would you consider participating in this clinical trial?**

a. Definitely would consider it

a. Probably would consider it

b. Probably would NOT consider it

c. Definitely would NOT consider it

**CONS**

**2c**. **Please select all the reasons that might decrease your willingness to participate in a clinical trial** ***(Select all that apply)***

a. It would take too much time or be inconvenient

b. I think medical research is unsafe

c. My family would be against it

d. I am worried it would have a bad side effect

e. It goes against my religious beliefs

f. I would not be able to choose which treatment I would get

g. I don’t think there are any benefits of participating in a research study

h. I don’t think that measures are in place to protect people like me

i. I participated in a research study in the past and had a bad experience.

j. Other: ___________________

**PROS**

**2d**.  **Please select all the reasons that might increase your willingness to participate in a clinical trial** ***(Select all that apply)***

a. It would give me hope for a better outcome

b. My family would want me to take part

c. It would help people like me who have the same condition in the future

d. I want to be sure that people like me are represented in clinical trials

e. I think people on clinical trials get better medical care

f. I would go along with what my doctor thinks is best

g. Other: ___________________

**3.** **How likely are you to encourage others (for example, community members like you or family members) to participate in a research study?**

a. Definitely would recommend it

b. Probably would recommend it

c. Probably would NOT recommend it

d. Definitely would NOT recommend it

**KNOWLEDGE ABOUT RESEARCH PROCESSES AND IRB:**

**4**. **We are interested in understanding what you know about clinical research practice requirements. Select TRUE or FALSE for each of the following statements:**

| Every research study has a protocol in place to protect the privacy of my information | **TRUE** | **FALSE** |
| --- | --- | --- |
| A review board (Institutional Review Board -IRB) reviews every study prior to approval to made sure that participant’s rights and safety will be protected | **TRUE** | **FALSE** |
| Clinical trials are closely monitored by the IRB and the lead researcher needs to tell the IRB each time a research participant has a medical problem. | **TRUE** | **FALSE** |
| If the review board sees that research participants are having unexpected or serious problems, studies may be stopped early. | **TRUE** | **FALSE** |
| If there is enough evidence that people are benefiting, the review board may end the study early so others can benefit from its findings. | **TRUE** | **FALSE** |
| The federal government has agencies that continuously monitor every clinical trial in the U.S. | **TRUE** | **FALSE** |
| Patients can decide to leave a research study at any time | **TRUE** | **FALSE** |
| Whether or not a patient has access to clinical trials depends upon where they go for health care | **TRUE** | **FALSE** |
| Hospitals and doctors in your neighborhood are involved in clinical trials | **TRUE** | **FALSE** |
| In order to receive federal funding, clinical trials are required to have a plan for ensuring that people participating in research studies represent the diversity of the US population. | **TRUE** | **FALSE** |

| **Trust in Research:**  **5.** **We are interested in understanding who you trust most when it comes to your health** | | | | | |
| --- | --- | --- | --- | --- | --- |
|  | **Strongly Disagree** | **Disagree** | **Neither Disagree nor Agree** | **Agree** | **Strongly Agree** |
| When it comes to my health, I believe **people like me in my community** have my best interest in mind |  |  |  |  |  |
| When it comes to my health, I believe **my friends and family** have my best interest in mind |  |  |  |  |  |
| When it comes to my health, I believe **my healthcare provider (doctor, nurse practitioner)** has my best interest in mind |  |  |  |  |  |
| When it comes to my health, I believe **scientists and researchers** have my best interest in mind |  |  |  |  |  |
| When it comes to my health, I believe the **healthcare care system** has my best interest in mind |  |  |  |  |  |
| When it comes to my health, I believe **pharmaceutical (drug) companies** have my best interest in mind |  |  |  |  |  |
| I have more trust in research studies now than before the COVID pandemic |  |  |  |  |  |
|  |  |  |  |  |  |
| **Trust in Research – participation, safety and outcomes**  **6.** **We are interested in understanding where you go to get trusted science information.** | | | | | |
|  | **Strongly Disagree** | **Somewhat Disagree** | **Neither Disagree nor Agree** | **Somewhat Agree** | **Strongly Agree** |
| I rely on my doctor and medical team for science information that I trust |  |  |  |  |  |
| I rely on the TV and news to learn about science and research studies |  |  |  |  |  |
| I trust that the scientific information shared on websites and media is accurate |  |  |  |  |  |
| I rely on social media to learn about science and research studies |  |  |  |  |  |
| I know how to identify if the health information I see on social media comes from a trusted source |  |  |  |  |  |
|  |  |  |  |  |  |
| **Trust in Research – Behavior around engagement**  **7.** **We are interested in understanding how more people like you could be recruited to participate in research** | | | | | |
|  | **Strongly Disagree** | **Disagree** | **Neither Disagree nor Agree** | **Agree** | **Strongly Agree** |
| I feel confident in my ability to explain to a friend what a research study is |  |  |  |  |  |
| I believe it is important for people like me, to participate in research |  |  |  |  |  |
| I believe clinical research helps save lives |  |  |  |  |  |
| I would join a community-based group to provide feedback on research studies |  |  |  |  |  |
| I would trust research more if I knew people like me were included on the research team |  |  |  |  |  |
| I would trust research more if I knew people like me were included as study participants |  |  |  |  |  |
| People like me should not trust medical researchers |  |  |  |  |  |
| People like me should be suspicious of information from medical researchers |  |  |  |  |  |
| People like me should be suspicious of medical research |  |  |  |  |  |
| Medical researchers treat people like me like guinea pigs |  |  |  |  |  |
| Medical researchers hide information from people like me |  |  |  |  |  |
|  |  |  |  |  |  |
| **What matters to you?**  **8.** **We are interested in understanding what matters to you related to participating in research studies** | | | | | |
|  | **Strongly Disagree** | **Disagree** | **Neither Disagree nor Agree** | **Agree** | **Strongly Agree** |
| I would feel more motivated to participate in a research study if I was paid fairly for my participation |  |  |  |  |  |
| If I knew that community members were involved in the design of research studies, I would trust the research more |  |  |  |  |  |
| It is important that scientists share what was learned from the research with participants |  |  |  |  |  |
| It is important that scientists share what was learned with the community |  |  |  |  |  |
| I would be more motivated to participate in a research study if the research team included people with the same race/ethnicity as me |  |  |  |  |  |
| I would be more motivated to participate in a research study if I know that the study went through a review board that made sure that participant’s rights and safety were protected before the study was approved. |  |  |  |  |  |
| I would be more motivated to participate in a research study if the study coordinator was empathetic, caring, and trustworthy |  |  |  |  |  |

**9.** **How much do you agree or disagree with each of the following statements about research?**

|  | **Strongly disagree** | **Somewhat disagree** | **Somewhat agree** | **Strongly agree** |
| --- | --- | --- | --- | --- |
| Medical research can help improve the care and services people receive. |  |  |  |  |
| Medical research is a way for the government to track down people they don’t want in the U.S. |  |  |  |  |
| The government should spend more money on research that finds cures and better treatments. |  |  |  |  |
| Research results are used to promote negative stereotypes and create more discrimination |  |  |  |  |
| Information that people give to researchers can be used against them. |  |  |  |  |
| There are enough safeguards to protect people who take part in medical research |  |  |  |  |

**10.** **The above questions above, the term “people like me” means:**

**Someone who . . . . .(select all that apply)**

a. is from the same racial/ethnic background as me

b. is from the same neighborhood or community as me

c. speaks the same language as me

d. is the same gender as me

e. is the same age as me

f. is from the same religion/faith as me

g. is a member of the LGBTQ+ community

h. Other: __________

**11.** **Research trials test new treatments to determine if they are effective. Patients who have failed all approved treatment options for their illness, may view enrolling in a clinical trial as something that might work when there is nothing else. What is your opinion about the following statements below?**

|  | **Strongly disagree** | **Disagree** | **Neither agree nor disagree** | **Agree** | **Strongly agree** |
| --- | --- | --- | --- | --- | --- |
| Patients should understand medical research in general even before they come down with an illness |  |  |  |  |  |
| Patient should be told about medical research immediately after diagnosis, even if it is only an option that might become relevant in the future. |  |  |  |  |  |
| Patients should be given the option of taking part in medical research instead of the standard course of therapy whenever possible. |  |  |  |  |  |
| Patients should only be told about medical research after all other treatment options have been exhausted |  |  |  |  |  |
| It is not appropriate to involve patients in medical research |  |  |  |  |  |

**12. Demographic Information**

**a.** **Age**

i. 18-25

ii. 26-35

iii. 36-45

iv. 46-55

v. 56-65

vi. 65-75

vii. 75-85

viii. 85+

**b.**  **Gender Identity**

i. Male

ii.  Female

iii. Transgender

iv. Other: _________

**c.** **Which of the following best describes your sexual orientation?**

i. Gay or lesbian

ii. Straight

iii. Bisexual

iv. I don’t know

v. Other: ______

**d. RACE and ethnicity: Which one or more of the following would you say is your race? (Select all that apply)**

i. White

ii. Black or Black American **→**  Skip to iia

**iia. Some people in addition to being Black, have a certain heritage or ancestry. Do you identify with any of these?**

a. African American

b. Caribbean or West Indian

c. A recent immigrant or the child of recent immigrants from Africa

d. Other

**e.** **What is your race and ethnicity? (Please check all that apply)**

i. American Indian, Alaska Native, First Nations, or Indigenous Peoples of the Americas

ii. Black or African American **→** Skip to iia

**iia. Some people in addition to being Black, have a certain heritage or ancestry. Do you identify with any of these?**

a. African American

b. Caribbean or West Indian

c. A recent immigrant or the child of recent immigrants from Africa

d. Other

iii. Hispanic or Latino/a **→** Skip to iiia

**iiia. Please tell us which group best represents your Hispanic heritage or ancestry? (Select all that apply)**

a. Mexican

b. Puerto Rican

c. Cuban

d. Dominican Republic

e. Central American

f. South American

g. Other

iv. Asian **→** Skip to iva

**iva. Please tell me which group best represents your Asian heritage or ancestry?**

a. Chinese

b. Asian Indian

c. Filipino

d. Korean

e. Japanese

f. Vietnamese

g. Other

 vi. Middle Eastern or North African

vii. Native Hawaiian/Pacific Islander

viii. White/Caucasian

ix. Other, please specify: _______

**f.** **Zip code: ___________**

**g.** **Sense of financial security: Which one of these comes closest to your own feelings about your household’s income these days?**

i. Living comfortably on present income

ii. Getting by on present income

iii. Finding it difficult on present income

iv. Finding it very difficult on present income

**h.** **Level of education: What is the highest grade or level of schooling you completed?**

i. Less than high school

ii. High school graduate/GED

iii. Some college

iv. College graduate (for example community college, bachelors)

v. Post-graduate degree (for example Master’s, MD, PhD)

**i.** **Gross Income**

**Thinking about yourself, members of your family and others living in your household , what is your combined annual income (total pre tax from all sources) earned in the past year?**

i. I/my family have no income

ii. Less than $20,000

iii. $20,000 to $34,999

iv. $35,000 to $49,999

v. $50,000 to $74,999

vi. $75,000 to $99,999

vii. Over $100,000

**j. How many people live in your household? Textbox response. ______**

**k.** **Disabilities: Select all that apply to you:**

i. I have NO physical, mental or emotional disabilities

ii. I am deaf or have serious difficulty hearing

iii. I am blind or have serious difficulty seeing, even when wearing glasses

iv. I have serious difficulty walking or climbing stairs

v. I have difficulty bathing or dressing

vi. Because of a physical, mental, or emotional problem, I have difficulty doing errands alone such as visiting a doctor’s office or shopping

vii. Because of a physical, mental, or emotional problem, I have difficulty remembering, concentrating, or making decisions

viii. I have a physical, mental or emotional disability, but do not have any difficulty independently completing errands or my daily activities

**POST CONVERSATION BRAID CIRCLE FEEDBACK FORM**

**(Administer After Each BRAID Session)**

|  | **Strongly Disagree** | **Disagree** | **Neither Disagree nor Agree** | **Agree** | **Strongly Agree** |
| --- | --- | --- | --- | --- | --- |
| **1.** During the meeting I felt comfortable voicing my point of view and concerns? |  |  |  |  |  |
| 2. During the meeting I felt respected as a subject matter expert in *what matters* most to my community? |  |  |  |  |  |
| 3. During the meeting I felt that my opinions and experiences were important |  |  |  |  |  |
| 4. During the meeting I felt like my concerns were addressed and my questions were answered |  |  |  |  |  |
| 5. My participation in today’s BRAID conversation has strengthened my trust in science? |  |  |  |  |  |
| 6. My participation in today’s BRAID conversation has strengthened my trust my care team? |  |  |  |  |  |
| 7. My participation in today’s BRAID conversation circle have made me more willing to encourage people like me to participate in research studies |  |  |  |  |  |
| 8. I will likely share something I learned or experienced today with someone else? |  |  |  |  |  |

**9.** **What other agenda items (discussion topics) would you recommend for future meetings?**

Describe here: __________

**10.** **Please share what can be done to improve the next BRAID circle, if anything: ______**

**EXPERIENCE PARTICIPATING IN BRAID**

**1.** **Did participating in BRAID conversation circles strengthen your trust in science and research?**

**2.** **What features about participating in BRAID circles strengthened your trust in science and research?** **(select all that apply)**

a. Having a chance to hear from scientists directly

b. Learning about the importance of having people like me participate in research studies

c. Learning about health disparities that affect my community

d. The data that was shared

e. Having the ability to ask questions to the people that conduct research studies

f. Having the opportunity to have a casual dialogue with researchers and health professionals

g. When researchers and doctors shared their own personal experiences it made them more human

h. The respectful way the researchers and doctors spoke with me as an equal

i. Other______

**3.** **Select all that apply: I shared information I learned or experienced during the series of conversation circles with:**

a. Other people like me

b. Members of my family

c. Friends and Neighbors

d. Community Leaders

e. Others ________________

**4.** **Would you encourage other people like you to participate in a BRAID conversation circle?**

a. Definitely would recommend it

b. Probably would recommend it

c. Probably would NOT recommend it

d. Definitely would NOT recommend it

5. **We are interested in understanding people’s attitudes about clinical trials. Clinical trials are studies where new medical treatments or therapies are tested.**

***Let’s say that you have a medical condition, and there is a clinical trial to test a new treatment that may or may not be better than current treatments for the health condition you have****.*

**If you were asked today, would you consider participating in this clinical trial?**

a. Definitely would consider it

b. Probably would consider it

c. Probably would NOT consider it

d. Definitely would NOT consider it

**CONS**

**5a. Please select all the reasons that might decrease your willingness to participate in a clinical trial *(Select all that apply)***

a. It would take too much time or be inconvenient

b. I think medical research is unsafe

c. My family would be against it

d. I am worried it would have a bad side effect

e. It goes against my religious beliefs

f. I would not be able to choose which treatment I would get

g. I don’t think there are any benefits of participating in a research study

h. I don’t think that measures are in place to protect people like me

i. I participated in a research study in the past and had a bad experience.

j. Other: ___________________

**PROS**

**5b.**  **Please select all the reasons that might increase your willingness to participate in a clinical trial** ***(Select all that apply)***

a. It would give me hope for a better outcome

b. My family would want me to take part

c. It would help people like me who have the same condition in the future

d. I want to be sure that people like me are represented in clinical trials

e. I think people on clinical trials get better medical care

f. I would go along with what my doctor thinks is best

g. Other: ___________________

**6.** **How likely are you to encourage others (for example, community members like you or family members) to participate in a research study?**

a. Definitely would recommend it

b. Probably would recommend it

c. Probably would NOT recommend it

d. Definitely would NOT recommend it

**KNOWLEDGE ABOUT RESEARCH PROCESSES AND IRB:**

7. **We are interested in understanding what you know about clinical research practice requirements. Select TRUE or FALSE for each of the following statements:**

| Every research study has a protocol in place to protect the privacy of my information | **TRUE** | **FALSE** |
| --- | --- | --- |
| A review board (Institutional Review Board -IRB) reviews every study prior to approval to made sure that participant’s rights and safety will be protected | **TRUE** | **FALSE** |
| Clinical trials are closely monitored by the IRB and the lead researcher needs to tell the IRB each time a research participant has a medical problem. | **TRUE** | **FALSE** |
| If the review board sees that research participants are having unexpected or serious problems, studies may be stopped early. | **TRUE** | **FALSE** |
| If there is enough evidence that people are benefiting, the review board may end the study early so others can benefit from its findings. | **TRUE** | **FALSE** |
| The federal government has agencies that continuously monitor every clinical trial in the U.S. | **TRUE** | **FALSE** |
| Patients can decide to leave a research study at any time | **TRUE** | **FALSE** |
| Whether or not a patient has access to clinical trials depends upon where they go for health care | **TRUE** | **FALSE** |
| Hospitals and doctors in your neighborhood are involved in clinical trials | **TRUE** | **FALSE** |
| In order to receive federal funding, clinical trials are required to have a plan for ensuring that people participating in research studies represent the diversity of the US population. | **TRUE** | **FALSE** |

| **Trust in Research:**  **8. We are interested in understanding who you trust most when it comes to your health** | | | | | |
| --- | --- | --- | --- | --- | --- |
|  | **Strongly Disagree** | **Disagree** | **Neither Disagree nor Agree** | **Agree** | **Strongly Agree** |
| When it comes to my health, I believe **people like me in my community** have my best interest in mind |  |  |  |  |  |
| When it comes to my health, I believe **my friends and family** have my best interest in mind |  |  |  |  |  |
| When it comes to my health, I believe **my healthcare provider (doctor, nurse practitioner)** has my best interest in mind |  |  |  |  |  |
| When it comes to my health, I believe **scientists and researchers** have my best interest in mind |  |  |  |  |  |
| When it comes to my health, I believe the **healthcare care system** has my best interest in mind |  |  |  |  |  |
| When it comes to my health, I believe **pharmaceutical (drug) companies** have my best interest in mind |  |  |  |  |  |
| I have more trust in research studies now than before the COVID pandemic |  |  |  |  |  |
| Participation in BRAID conversation circles increased my trust in research and science |  |  |  |  |  |
|  |  |  |  |  |  |
| **Trust in Research – participation, safety and outcomes**  **9. We are interested in understanding where you go to get trusted science information.** | | | | | |
|  | **Strongly Disagree** | **Somewhat Disagree** | **Neither Disagree nor Agree** | **Somewhat Agree** | **Strongly Agree** |
| I rely on my doctor and medical team for science information that I trust |  |  |  |  |  |
| I rely on the TV and news to learn about science and research studies |  |  |  |  |  |
| I trust that the scientific information shared on websites and media is accurate |  |  |  |  |  |
| I rely on social media to learn about science and research studies |  |  |  |  |  |
| I know how to identify if the health information I see on social media comes from a trusted source |  |  |  |  |  |
|  |  |  |  |  |  |
| **Trust in Research – Behavior around engagement**  **10. We are interested in understanding how more people like you could be recruited to participate in research** | | | | | |
|  | **Strongly Disagree** | **Disagree** | **Neither Disagree nor Agree** | **Agree** | **Strongly Agree** |
| I feel confident in my ability to explain to a friend what a research study is |  |  |  |  |  |
| I believe it is important for people like me, to participate in research |  |  |  |  |  |
| I believe clinical research helps save lives |  |  |  |  |  |
| I would join a community-based group to provide feedback on research studies |  |  |  |  |  |
| I would trust research more if I knew people like me were included on the research team |  |  |  |  |  |
| I would trust research more if I knew people like me were included as study participants |  |  |  |  |  |
| People like me should not trust medical researchers |  |  |  |  |  |
| People like me should be suspicious of information from medical researchers |  |  |  |  |  |
| People like me should be suspicious of medical research |  |  |  |  |  |
| Medical researchers treat people like me like guinea pigs |  |  |  |  |  |
| Medical researchers hide information from people like me |  |  |  |  |  |
|  |  |  |  |  |  |
| **What matters to you?**  **11. We are interested in understanding what matters to you related to participating in research studies** | | | | | |
|  | **Strongly Disagree** | **Disagree** | **Neither Disagree nor Agree** | **Agree** | **Strongly Agree** |
| I would feel more motivated to participate in a research study if I was paid fairly for my participation |  |  |  |  |  |
| If I knew that community members were involved in the design of research studies, I would trust the research more |  |  |  |  |  |
| It is important that scientists share what was learned from the research with participants |  |  |  |  |  |
| It is important that scientists share what was learned with the community |  |  |  |  |  |
| I would be more motivated to participate in a research study if the research team included people with the same race/ethnicity as me |  |  |  |  |  |
| I would be more motivated to participate in a research study if I know that the study went through a review board that made sure that participant’s rights and safety were protected before the study was approved. |  |  |  |  |  |
| I would be more motivated to participate in a research study if the study coordinator was empathetic, caring, and trustworthy |  |  |  |  |  |

**12. How much do you agree or disagree with each of the following statements about research?**

|  | **Strongly disagree** | **Somewhat disagree** | **Somewhat agree** | **Strongly agree** |
| --- | --- | --- | --- | --- |
| Medical research can help improve the care and services people receive. |  |  |  |  |
| Medical research is a way for the government to track down people they don’t want in the U.S. |  |  |  |  |
| The government should spend more money on research that finds cures and better treatments. |  |  |  |  |
| Research results are used to promote negative stereotypes and create more discrimination |  |  |  |  |
| Information that people give to researchers can be used against them. |  |  |  |  |
| There are enough safeguards to protect people who take part in medical research |  |  |  |  |

**13. In the questions above, the term “people like me” means:**

**Someone who . . . . .(select all that apply)**

a. is from the same racial/ethnic background as me

b. is from the same neighborhood or community as me

c. speaks the same language as me

d. is the same gender as me

e. is the same age as me

f. is from the same religion/faith as me

g. is a member of the LGBTQ+ community

h. Other: __________

**14. Taking part in medical research (clinical trial) may become a treatment option for some patients. What is your opinion about the following statements below:**

|  | **Strongly disagree** | **Disagree** | **Neither agree nor disagree** | **Agree** | **Strongly agree** |
| --- | --- | --- | --- | --- | --- |
| Patients should understand medical research in general even before they come down with an illness |  |  |  |  |  |
| Patient should be told about medical research immediately after diagnosis, even if it is only an option that might become relevant in the future. |  |  |  |  |  |
| Patients should be given the option of taking part in medical research instead of the standard course of therapy whenever possible. |  |  |  |  |  |
| Patients should only be told about medical research after all other treatment options have been exhausted |  |  |  |  |  |
| It is not appropriate to involve patients in medical research |  |  |  |  |  |

**Appendix C**

**Getting Ready to BRAID Assessment Tool**

**I. BACKGROUND INFORMATION**

Name: Title/Current Position:

Affiliated Organization: Length of Time at Organization:

**II. REFLECTION & FEEDBACK**

1. Which aspects of the BRAID experience did you find most meaningful? Why?

2. Which aspects of the BRAID experience do you think could be improved?

3. What did you learn from the BRAID experience?

**III. BRAID READINESS (ADKAR MODEL OF CHANGE)**

Please choose the most accurate response to the following statements regarding your individual experience with BRAID.

**When it comes to sharing messages about the importance of diversity in clinical trials:**

**1.** **AWARENESS**: How aware are you about the reasons that diversity in clinical trials is important?

| Not at All  (1) | Slightly  (2) | Moderately  (3) | Very  (4) | Extremely  (5) |
| --- | --- | --- | --- | --- |

**2.** **DESIRE:** How motivated are you to share information about clinical trials to your community (family, friends, peers, faith-based groups, community groups etc.)?

| Not at All  (1) | Slightly  (2) | Moderately  (3) | Very  (4) | Extremely  (5) |
| --- | --- | --- | --- | --- |

**3.** **KNOWLEDGE:** How confident are you that you have the knowledge needed to share accurate messages about the importance of including people from your community in clinical trials?

| Not at All  (1) | Slightly  (2) | Moderately  (3) | Very  (4) | Extremely  (5) |
| --- | --- | --- | --- | --- |

**4.** **ABILITY:** How confident are you that you have the tools and materials (videos, website links, social media messages) needed to easily share accurate messages about clinical trials to your community?

| Not at All  (1) | Slightly  (2) | Moderately  (3) | Very  (4) | Extremely  (5) |
| --- | --- | --- | --- | --- |

*a.* What additional information and resources would be helpful? Indicate the tools that would support your success in engaging other community members in conversation.

- Handouts/flyers
- Educational Videos
- Translated materials
- Communication training on how to BRAID
- Other: _______________________________________________________________

5. **REINFORCEMENT:**

1. How ready do you think people in your community will be to learn this information?

| Not at All  (1) | Slightly  (2) | Moderately  (3) | Very  (4) | Extremely  (5) |  |
| --- | --- | --- | --- | --- | --- |

b. How receptive do you think that your community will be to the available messages and resources that were shared?

| Not at All  (1) | Slightly  (2) | Moderately  (3) | Very  (4) | Extremely  (5) |
| --- | --- | --- | --- | --- |

i. [PROBE FURTHER IF RESPONSE 1-4] What kinds of modifications could make the materials more appropriate for your community?

|  |
| --- |

c. How much do you think that people in your community will appreciate that you are volunteering your time to share information about clinical trials with them?

| Not at All  (1) | Slightly  (2) | Moderately  (3) | Very  (4) | Extremely  (5) |  |
| --- | --- | --- | --- | --- | --- |

d. How strongly do you feel that people in your community see you as a trusted messenger of accurate health information?

| Not at All  (1) | Slightly  (2) | Moderately  (3) | Very  (4) | Extremely  (5) |
| --- | --- | --- | --- | --- |

**Select the best response:**

*I plan to continue the conversation and share accurate information about clinical trials downstream in my social network.*

A. I have already done this and will continue

B. I will do this

C. I might do this

D. I want to do this, but I don’t feel ready/confident yet

E. I won’t do this

[IF RESPONSE C, D OR E IS SELECTED, THEN PROBE DEEPER TO UNDERSTAND WHAT WOULD HELP THEM FEEL MORE CONFIDENT OR WILLING]

i. What do you need to feel more confident or willing?

· Communication training

· Different types of materials

· Greater depth of information

· A central place where I can easily get messages to share

o What would that look like?

· Other _________________________________________

**AWARENESS: How much did participation in the BRAID conversation circles contribute to**

**1.** Your awareness of why diversity in clinical trials is important?

| Not at All  (1) | Slightly  (2) | Moderately  (3) | Very  (4) | Extremely  (5) |
| --- | --- | --- | --- | --- |

**DESIRE:** **How much did participation in the BRAID conversation circles contribute to**

**2.** Your motivation to share information about clinical trials to your community (family, friends, peers, faith-based groups, community groups etc.)?

| Not at All  (1) | Slightly  (2) | Moderately  (3) | Very  (4) | Extremely  (5) |
| --- | --- | --- | --- | --- |

**KNOWLEDGE:** **How much did participation in the BRAID conversation circles contribute to**

**3.** Your confidence that you have the knowledge needed to share accurate messages about clinical trials to your community?

| Not at All  (1) | Slightly  (2) | Moderately  (3) | Very  (4) | Extremely  (5) |
| --- | --- | --- | --- | --- |

**ABILITY:** **How much did participation in the BRAID conversation circles contribute to**

**4.** Your ability to easily share accurate messages about clinical trials to your community, utilizing tools and messages that you helped co-create or were shared with you?

| Not at All  (1) | Slightly  (2) | Moderately  (3) | Very  (4) | Extremely  (5) |
| --- | --- | --- | --- | --- |

**REINFORCEMENT:** **How much did your participation in BRAID conversations ...**

5. Reinforce your belief that increasing diversity in clinical trials matters to your community?

| Not at All  (1) | Slightly  (2) |  | Moderately  (3) | Very  (4) | Extremely  (5) |
| --- | --- | --- | --- | --- | --- |

6. Build your trust in science and the importance of diversity in clinical trials?

| Not at All  (1) | Slightly  (2) | Moderately  (3) | Very  (4) | Extremely  (5) |
| --- | --- | --- | --- | --- |

7. Build your trust in the motives of academic researchers to recruit people from your community into clinical trials?

| Not at All  (1) | Slightly  (2) | Moderately  (3) | Very  (4) | Extremely  (5) |
| --- | --- | --- | --- | --- |

**IV. BRAID COMMUNITY EXPERT NETWORK** [FORMATTED IN EXCEL WITH DROPDOWNS]

Please indicate eight (8) or more community members you plan to share information with.

| **Initials** | **Relationship to Self**    **(Family, coworker, neighbor, friend, church/faith member, physician/PCP, other)** | **Title/Occupation**    **(Educator, health worker, clergy, caregiver, other)** | **Demographic Description**    **(Age, gender, racial/ethnic identity)** | **Reason for Engagement**    **(Current chronic illness, caregiver, other)**    (Why is this a good person to learn about the message from BRAID?) | **How much do they trust you as a bearer of information?**    **(Not at all, Slightly, Moderately, Very, Extremely)** | **Anticipated Challenges**    (How receptive do you think this person will be to the message?) |
| --- | --- | --- | --- | --- | --- | --- |

[AFTER FOLLOW UP IN 4-6 WEEKS] Please share your experiences engaging the above individuals.

| **Initials** | **Timing of Engagement**    **(Immediately after BRAID circles, 1 week after, etc.)** | **Continuity of Outreach** | **Total Number of Follow Ups** | **Response Received/Outcome**    **(Not available, not interested in topic, positive response, interested in additional information, other - please specify)** |
| --- | --- | --- | --- | --- |

**V. IDENTIFYING FUTURE BRAIDERS**

6. Please share the names and contact information of community members you have already contacted and would recommend for participation in future BRAID conversation circles on different content areas.

| **Name** | **Title** | **Contact Information**  (phone, email) | **Content Expertise**  (Dementia, cancer, etc.) |
| --- | --- | --- | --- |
|  |  |  |  |
|  |  |  |  |
|  |  |  |  |

7. Can we contact you to participate in future BRAID conversation circles? ____Yes _____No

**VI. Additional Questions: (pages 7-12)**

**FEEL SAFE TO SHARE**

**1.** **At what point during your engagement with the BRAID team did you first feel that the space that was created was safe enough for you to openly express your thoughts about clinical trials?**

***Can probe:***

1. During my initial recruitment conversation (skip pattern 1a)

2. During the research consent process (skip pattern 1a)

3. During Conversation Circle #1 where we were first oriented to the BRAID model and conversation circle structure and goals (skip pattern 1a)

4. During Conversation Circle #2. (skip pattern 1a)

5. During Conversation Circle #3. (skip pattern 1a)

6. During Conversation Circle #4. (skip pattern 1a)

7. During the **Getting Ready to BRAID Session** where BRAID resources and MI communication skills to support me in my BRAIDer role were shared. (skip pattern 1a)

8. I never felt safe enough to share (skip pattern to 1b)

1a. What made you feel safe? _________________________

Drop down options multi-select (we need to develop drop down list to ease the data collection burden)

· The facilitator

· The fact that I felt respected

· Permission was asked

· The facilitator modeled MI!

· The facilitators were like me.??

· The members in the group made me comfortable

1b. If none, what could have been done to make you feel safe? ______________________________

**ENOUGH INFORMATION**

**2.** **Do you believe sufficient information was provided during the four (4) Conversation Circle meetings and Getting Ready to BRAID session to prepare you to START educating and sharing what you learned with others?**

**Yes No**

2a. If No,

What other information or resources are needed? __________________________

*(can create drop downs based on the kinds of responses received to add ease to data collection and analysis)*

**TRUST: Quantitative**

**3.** **On a scale of 1 to 10 where was your trust in scientific research and clinical trials before you participated in BRAID? _____**

1 2 3 4 5 6 7 8 9 10

No Trust 100% Trust

**4.** **On a scale of 1 to 10 where is your trust in scientific research and clinical trials NOW? ______**

1 2 3 4 5 6 7 8 9 10

No Trust 100% Trust

**Self- Efficacy: BRAIDing ABILITY - Skills and Knowledge Confidence**

**5.** ***On a scale of 1-5 where 1 is I don’t feel ready or confident and 5 is I am very ready or confident. .. . .***

**How ready or confident are you that you have the knowledge and skills needed to begin BRAIDing (the process of sharing accurate information about clinical trials with others)?**

1 2 3 4 5

not at all ready slightly moderately very extremely

*For choices 2,3,4,5 ask 4a*

5a. Why did you select a “X (i.e 2)” rather than a “Y” (lower number (i.e. 1)? __________________-

*For choices 1,2,3,4 ask 4b*

5b. What would move you from a “X” (the selected number (i.e.2) to a “Y” (higher number (i.e. 3) in readiness? ________________

5c. What additional knowledge or skills training would be helpful? ______________________________

**Getting Ready to BRAID meeting**

**6.** **Which agenda items at the “Getting Ready to BRAID meeting was the most valuable to you?**

· Discussing the Conversation Circles meetings and the impact it had on trust and attitudes toward clinical trials

· Sharing your experience engaging community members

· Reviewing and contributing to the development of the FAQ

· Motivational Interviewing presentation and coaching

**MI TRAINING**

**7.** **How helpful do you think the Motivational Interviewing training and MI tip sheet that Dr. Gutnick provided will be to help you have effective conversations about research and clinical trials with members of your community?**

1 2 3 4 5

not at all valuable slightly moderately very extremely valuable

7a. What other communication skills or MI training or resources would be helpful? __________________________

· Additional Training Program with time for role playing practice and coaching and feedback

· Time to practice teach back skills

· The opportunity to follow-up with the BRAID team if I have questions and or need advice

· Other

7b. If additional MI training were offered to the BRAID community, would you attend?

· YES Definitely

· YES, only if I were paid for participating

· NO

· Not sure

7c. Would you recommend MI training to other trusted messengers in your community?

YES. NO Not sure

**Modeling MI**

**The BRAID facilitators strived to model Motivational Interviewing during the BRAID circles, (for example: asked permission before sharing information, respected your autonomy, encouraged you to share your ideas etc.) Reflecting on your experience with BRAID did you feel the impact of the spirit of Motivational Interviewing?**

YES NO NOT SURE

8a. If yes, did this contribute to your trust of the BRAID process?

YES NO Not sure

8b. Do you feel that this contributed to the trust that was built within the group?

**Self- Efficacy MI ABILITY**

***On a scale of 1-5 where 1 is I don’t feel ready or confident and 5 is I am very ready or confident,***

**9. How confident do you feel about using Motivational Interviewing skills and concepts during BRAIDing conversations?**

1 2 3 4 5

not at all confident slightly moderately very extremely confident

What would increase your confidence? ______________

**BRAID Participation Recommendation**

**10. Would you recommend that other trusted messengers in your community participate in the BRAID program?**

YES NO Not sure

**Appendix D**

**Conversation Circle Tasks and Roles**

The following tables contain the tasks and roles required to conduct BRAID conversation circles on Zoom and in-person. Keep in mind that these tasks/roles are suggested, and that one person can fulfill more than one role so long as the assigned roles do not conflict. For instance, the Technology Production and Facilitator role should ideally not be shared by one person. The Circle Facilitator and Moderator/’s can divide roles that make sense for the size of the group and meeting modality. *Also see checklist tool in Appendix*

**Virtual (Zoom) Conversation Circle Tasks and Roles**

| **Task/Role**  **Who will do this?** | **During Conversation Circle** | **After/Between Conversation Circles** |
| --- | --- | --- |
| **Facilitator** | - Host meeting and lead introductions of team and participants - Ensure that participants are engaged | - Coordinate with health system to identify scientific/medical experts for follow-up circles - Orient scientific and medical experts to circle guidelines and practices |
| **Technology Production** | - Monitor waiting room - Turn on recording when participant’s join - Change/fix participant and visitor names - Mute attendees when needed | - Download Zoom recording and chat and save into TEAMS folder - Clean and organize zoom recording transcript - Deidentify transcript |
| **Notetaker** | - collect observations about participants and key themes and quotes | - compare and consolidate notes with data and parking lot slide |
| **Parking Lot (PL) Manager** | - Capture the key questions and themes shared by the participants - Prepare parking lot document to display and review at end of session | - Summarize key themes and questions from parking lot - File parking lot slides for team review, sharing |
| **Consent Manager** *only if consent not obtained prior to first circle* | - Review consents from chat | - Sign and file oral consents and document in attendance tracker |
| **Survey Manager** | - Put survey links in chat and send survey links via email if needed - Ensure all participants complete pre survey and inform the facilitator if needed (Survey Monkey) | - Ensure participants complete post survey - Download survey reports for analysis |
| **Chat Monitor** | - Message participants and facilitator/team when needed - Manage attendee questions/requests and private chat facilitators as needed |  |
| **Attendance/ Participation Monitoring** | - Keep track of participation and hours – create excel file   *(See Appendix: Attendance tracker document)* | - Participant compensation management (i.e. gift cards) and reporting at conclusion of circle |
| **Timekeeper** | - Ensure circle starts and ends on time - Record (approximate) time for each circle component and timestamps for key thematic quotes | - co - Report on time spent during debrief (if needed) |
| **Co-Facilitator (optional)** | - Share slides from their screen - Review Circle Guidelines with group - Support facilitator |  |
| **Communication Manager** | - Send invite to participants - Confirm attendees with team | - Send follow up thank you email to participants - Send details for next meeting - Send zoom link for next meeting |

**In-Person Conversation Circle Tasks and Roles**

| **Task/Role**  **Who will do this?** | **During Conversation Circle** | **After/Between Conversation Circles** |
| --- | --- | --- |
| **Facilitator** | - Host meeting and lead introductions of team and participants - Ensure that participants are engaged | - Coordinate with health system to identify scientific/medical experts for follow-up circles - Orient scientific and medical experts to circle guidelines and practices |
| **Recorder** | - Set-up audio-recording device - Perform sound-check on recording device - Start the recording when participants join | - Download and save audio recording into TEAMs folder - Prepare recording for transcription |
| **Notetaker** | - collect observations about participants and key themes and quotes | - compare and consolidate notes with data and parking lot slide |
| **Parking Lot (PL) Manager** | - Capture the key questions and themes shared by the participants - Record expertise, materials participants are interested in flip chart - Prepare parking lot document to display and review at end of session (Flip chart) | - Summarize key themes and questions from parking lot (flip chart) - File parking lot slides for team review, sharing (take a photo of flip chart content ) |
| **Consent Manager** | - Conduct informed consent - Collect signed consents | - File completed consents |
| **Survey Manager** | - Share survey or send links to participants via email if needed or collect paper surveys - Ensure all participants complete pre survey (check Survey Monkey) | - Ensure participants complete post survey - Download survey reports for analysis |
| **Attendance/Participation Monitoring** | - Keep track of participation and hours – using attendance tracker excel file (see Appendix) | - Participant compensation management (i.e. gift cards) and reporting at conclusion of circle |
| **Timekeeper** | - Ensure circle starts and ends on time - Record (approximate) time for each circle component - Record time stamps of powerful quotes | - Identify time issues and share with team - Report on time spent during debrief (if needed) |
| **Communication Manager** | - Send invite to participants - Confirm attendees with team | - Send follow up thank you email to participants - Send details for next meeting |

**Appendix E**

**Sharing Circle Guidelines**

Conversation Circles are designed to build trust between community members and the health system. Therefore, it is important to share guidelines and expectations with all participants, before the conversation begins. The circle guidelines should be read aloud before every circle. The guidelines should also be displayed for participants to see, using a slide or a large board. Circle participants should be invited to add any additional guidelines that they feel are important. Guidelines recommended by participants should be added to the list.

The following script and guidelines can be used to facilitate circle discussion.


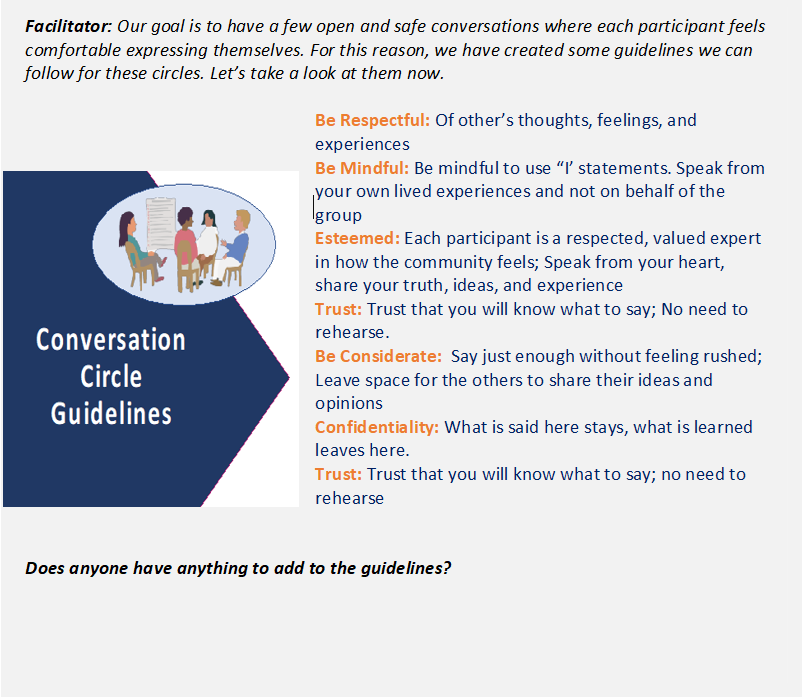

Supplement: Supplementary file 1 [file Data_Sheet_1.docx]
